# Necrology

**Published:** 1891-03

**Authors:** 


### NECROLOGY.

The veterinary profession will hear with deep regret of the death of John H. Steel, F. R. C. V. S., Army Veterinary Department, which occurred at Bombay, India, on January 20th.

Although only 35 years of age, Mr. Steel had accomplished through his phenomenal enthusiasm, distinguished ability, and untiring energy, a degree of success which will make his name one of the lasting monuments of the veterinary profession, and his promise, for the future, makes his loss not only a bereavement to his friends, but a catastrophy for veterinary science.

Mr. Steel graduated in 1875, and soon after joined the Army Veterinary Department; in this he only remained a short time, as he accepted an appointment on the teaching Staff of the Royal Veterinary College. This position, after a brief period, he found unsuitable and joined the army, soon afterward proceeding to India. Stationed at Bombay, he commenced a career of wonderful activity. Mainly through his influence the Bombay Veterinary School was established, and as Principal he was instrumental in making it a most useful and recognized educational establishment. By the authorities in the Bombay Government his services were highly appreciated and the annual reports of the School afford evidence that he well deserved all that he said of him by those who were made aware of what he had done and was doing for the presidency. In fact, Mr. Steel was a most valuable public servant outside the college, and in everything relating to animals he was consulted and valued as an authority. This caused him to be elected to discharge many functions beyond his own special duties. The Governor of Bombay, Lord Reay, appointed him a Justice of the Peace, and the Bombay University conferred upon him the honorary degree of Fellow.

His writings are numerous and well known. We may mention his "Outlines of Equine Anatomy," "Diseases of the Elephant," "Canine Pathology." "Bovine Pathology," and the quite recent "Treatise of the Diseases of the Sheep," a review of which will appear in the next number of the JOURNAL. He was also one of the Editors of the *Quarterly Journal of Veterinary Science*, in India (Mr. Fred. Smith, Army Veterinary Surgeon, being the other), and in this periodical appeared many valuable and interesting papers from his pen.

He was a Fellow of the Royal College of Veterinary Surgeons, and of the Zoological Society, and it was owing to his efforts that the National Veterinary Association was begun; he was its first Secretary.

